# Prevalence and pattern of antibiotic resistance of *Staphylococcus aureus* isolated from door handles and other points of contact in public hospitals in Ghana

**DOI:** 10.1186/s13756-017-0203-2

**Published:** 2017-05-10

**Authors:** Courage Kosi Setsoafia Saba, Jean Kwadwo Amenyona, Stephen Wilson Kpordze

**Affiliations:** 1 Department of Biotechnology, Faculty of Agriculture, University for Development Studies, P. O Box TL 1882, Tamale, Ghana; 2 Department of Ecotourism and Environmental Management, Faculty of Renewable Natural Resources, University for Development Studies, P. O Box TL 1882, Tamale, Ghana

**Keywords:** Healthcare associated infections, Methicillin resistant *Staphylococcus aureus*, Antibiotic resistance, Ghana

## Abstract

**Background:**

Studies have implicated *Staphylococcus aureus* as the leading cause of septicemia in the Tamale metropolis of Ghana*.* The aim of this study was to determine the prevalence and antibiotic susceptibility of *S. aureus* and Methicillin Resistant *S. aureus* (MRSA) in the environments of three hospitals in Ghana.

**Methods:**

A total of 120 swab samples were taken from door handles, stair railings and other points of contact at Tamale Teaching Hospital, Tamale Central Hospital and Tamale West Hospital. The swab samples were directly plated on Mannitol Salt and Baird Parker agar plates and incubated at 37 °C (± 2) for 18–24 h. An antibiotic susceptibility test was performed using the Clinical Laboratory Standard Institute’s guidelines. Isolates resistant to both cefoxitin and oxacillin were considered to be MRSA.

**Results:**

A total of 47 (39%) positive *S. aureus* samples were isolated from all three hospitals, of which, eight (17%) were putative MRSA isolates. One MRSA isolate was resistant to all the antibiotics used (cefoxitin, oxacillin, ciprofloxacin, erythromycin, tetracycline, ampicillin, streptomycin and sulfamethoxazole-trimethoprim). Five of the MRSA isolates were multi-drug resistant, whilst the other three were resistant to only two antibiotics. All the multi-drug resistant MRSA isolates were resistant to at least four antibiotics. The percentage of isolates resistant to oxacillin, ampicillin, ciprofloxacin, tetracycline, streptomycin, erythromycin, and sulfamethoxazole/trimethoprim were 17, 13, 9, 28, 89, 13 and 11% respectively.

**Conclusion:**

The high multi-drug resistance of MRSA in hospital environments in Ghana reinforces the need for the effective and routine cleaning of door handles in hospitals. Further investigation is required to understand whether *S. aureus* from door handles could be the possible causes of nosocomial diseases in the hospitals.

**Electronic supplementary material:**

The online version of this article (doi:10.1186/s13756-017-0203-2) contains supplementary material, which is available to authorized users.

## Background

Constant surveillance and adequate infection control measures for *Staphylococcus aureus* and Methicillin Resistant *Staphylococcus aureus* (MRSA) may reduce their roles in the incidence of nosocomial diseases and other infections in a clinical setting. However, there is a paucity of data *on S. aureus* prevalence and antibiotic susceptibility in low resource settings such as Ghana [1]. Most available data on *S. aureus* prevalence in Africa are patient-based, although the ‘one health’ concept (including environmental persistence) must be considered if this organism is to be effectively controlled. The ease of spread of resistant strains of *S. aureus* or MRSA cannot be overemphasized, especially in the Ghanaian context, where both environmental and personal hygiene are still rudimentary [2, 3]. *S. aureus* is known for its ability to develop resistance to almost all antibiotics, which makes it challenging to treat the range of infections it causes. There are reports of increasing resistance by MRSA and Methicillin Susceptible *S. aureus* (MSSA) to drugs of choice [4]. Very few published studies exist on investigating environmental samples as sources of possible nosocomial diseases in Ghana and in Africa in general. However, a study in the neonatal intensive care unit in Accra identified *S. aureus* as the predominant bacteria (44%) found in the hospital environment [5]. Similar work on swab samples in the hospital environment in Nigeria also recorded *S. aureus* as the most prevalent microbe (50.80%) in the hospital environment [6] and a study of door handles and bathtubs in the hospital environment in Benin implicated the Panton-Valentine Leukocidin (PVL) producing *S. aureus* as a cause of nosocomial diseases [7]. In the USA, current estimates indicate MRSA causes approximately 95,000 invasive infections and 19,000 mortality cases per year, which is higher than the mortality rates caused by AIDS/Human Immunodeficiency Virus, hepatitis, tuberculosis and influenza combined [8]. The prevalence of multi-drug resistant MRSA in clinical isolates has been reported to be very high in some African countries including Morocco, Kenya, Nigeria and Cameroon [9].

In the Tamale Metropolis of Ghana, *S. aureus* has been implicated in 60.9% of sepsis cases among children in the Tamale Teaching Hospital [10]. *S. aureus* was also reported to be the commonest contaminant found in donor blood in Tamale with the following resistance pattern: 71.5, 28.6, 71.5 and 10% to Ampicillin, Ciprofloxacin, Tetracycline and Erythromycin respectively [11]. In Ghana, *S. aureus* is the third most commonly isolated microbe from patients, after *E. coli* and *Pseudomonas* spp. [12], the second most prevalent bacteria among patients from teaching, regional and district hospitals with a multi-drug resistant rate of 42.3% [13], and the second most frequently isolated organism in bacteremia incidence in the Ashanti Region of Ghana [14].

The aim of this study was to determine the prevalence and antibiotic susceptibility of *S. aureus* and Methicillin Resistant *S. aureus* (MRSA) in the environments of three hospitals in Ghana. Specifically, the objectives of this study were to determine the prevalence of *S. aureus* and MRSA on the door handles, stair railings, tap handles and surgical room aprons in the environment of three hospitals, and to determine the antibiotic susceptibility levels of isolated *S. aureus* and MRSA.

## Methods

The study was carried out at the three major government hospitals in the Tamale Metropolis of Ghana: Tamale Teaching Hospital (TTH), is a referral hospital for three regions of Ghana with 478 beds and over 2000 workers; Tamale Central Hospital (TCH), is a public tertiary healthcare facility with 156 beds and 442 staff, and Tamale West Hospital (TWH), which is also a public tertiary healthcare center with 126 beds and 370 staff. The sample collection started in February and ended in March 2015. Permission and ethical clearance was granted by all three hospitals to conduct this research.

**Fig. 1:**
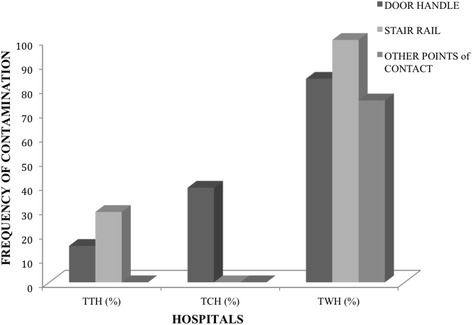
*S. aureus* prevalence at the various hospitals. TTH, Tamale Teaching Hospital, TCH, Tamale Central Hospital, TWH, Tamale West Hospital

Samples were randomly taken from 120 points of contact: 94 (78%) were taken from door handles, eight (7%) were from stair rails and 18 (15%) were from other points of contact (i.e. pipe taps, emergency beds, aprons in the theatre, and doors without handles) (Table 1). Out of the total 120 samples, 51 (43%) were taken from the TTH, 35 (29%) from TCH and 34 (28%) from TWH (Fig. 1). Individual sterile swabs (MEUS, Italy) moistened with sterile phosphate buffer saline (Oxoid, Hampshire, UK) were used to swab each points of contact. The samples were kept below 4 °C and transported to the laboratory for analysis within two hrs. All swab samples were then directly streaked onto Mannitol Salt agar (Oxoid) media and incubated at 37 °C for 24 h. Suspected *S. aureus* positive colonies were re-streaked on Baird Parker agar (BioMerieux S. A, Spain), and incubated at 37 °C for 24 h for confirmation*.* Pure cultures of *S. aureus* were plated onto nutrient agar (Oxoid,) prior to all susceptibility tests. Antibiotic susceptibility testing was performed and interpreted according to the Clinical and Laboratory Institute Standard (CLSI) guidelines [15]. The breakpoints were determined using National Committee for Clinical Laboratory Standards (NCCLS) document M31-A2 [16]. Oxacillin (1 μg), Ampicillin (25 μg), Ciprofloxacin (5 μg), Tetracycline (30 μg), Erythromycin (15 μg), Streptomycin (10 μg) and Sulphamethoxazole/Trimethoprim (25 μg) were used for the susceptibility tests (all anitbiotics obtained from BioMerieux). Cefoxitin disks were used to test only those isolates resistant to Oxacillin. Methicillin Resistant *S. aureus* strains were classified as those isolates resistant to Cefoxitin and Oxacillin. Multi-drug resistant isolates were classified as those resistant to three or more antibiotics. All isolates have been stored in 25% glycerol at −20 °C, and are available for future studies.

**Table 1:** *S. aureus* prevalence at the various departments/wards at each hospital

Department/Hospital	TTH (%)	TCH (%)	TWH (%)	Total
Administration	1/9 (11%)	1/3 (33%)	3/3 (100%)	5/15 (33%)
OPD	2/12 (17%)	2/7 (29%)	11/12 (92%)	15/31 (48%)
Theatre	0/13 (0%)	1/5 (20%)	7/10 (58%)	8/28 (29%)
Laboratory	2/6 (33%)	2/3 (67%)	–	4/9 (44%)
Male ward	–	0/2 (0%)	5/5 (100%)	5/7 (71%)
Female ward	–	2/2 (100%)	0/2 (0%)	2/4 (50%)
Children’s ward	0/2 (0%)	0/3 (0%)	–	0/5 (050
Child welfare centre	–	1/2 (50%)	–	1/2 (50%)
New born intensive care	–	0/2 (0%)	–	0/2 (0%)
Maternity	–	0/3 (0%)	–	0/3 (0%)
Festula department	–	2/3 (67%)	–	2/3 (67%)
X-ray	–	–	1/1 (100%)	1/1 (100%)
Aseptic ward	0/1 (0%)	–	–	0/1 (0%)
Male surgical ward	1/1 (100%)	–	–	1/1 (100%)
Elevator button	0/1 (0%)	–	–	0/1 (0%)
Stair rail	2/7 (29%)	–	1/1 (100%)	3/8 (38%)
Total	8/51 (16%)	11/35 (31%)	28/34 (82%)	47/120 (39%)

*TTH* Tamale Teaching Hospital, *TCH* Tamale Central Hospital, *TWH* Tamale West Hospital, *OPD* Out Patient Department

## Results

### Sources and isolation of *Staphylococcus aureus*

A total of 104 suspected small yellow colonies of *S. aureus* were isolated from Mannitol Salt agar plates, and of these 47 isolates *S aureus*, indicated by black colonies with clear zones, were confirmed on Baird Parker plates (Table 1).

### Antibiotic susceptibility of *S. aureus* isolates

Antibiotic susceptibility tests were performed on the 47 (39%) positive *S. aureus* samples. Of the total 47 positive *S. aureus* isolates, the percentage of isolates resistant to Oxacillin (OX), Ampicillin (AMP), Ciprofloxacin (CIP), Tetracycline (TET), Streptomycin (SMN), Erythromycin, (ERY) and Sulfamethoxazole/Trimethoprim (SXT) was 17, 13, 9, 28, 89, 13 and 11% respectively. Twenty eight (60%) isolates were isolated from the TWH alone, with the following resistance pattern; OX (18%), AMP (14%), CIP (14%), TET (46%), SMN (89%), ERY, (21%) and SXT (14%). One MRSA isolate from TWH was resistant to all the antibiotics used. Eleven (23%) isolates were from the TCH with the following resistance pattern: OX (18%), AMP (9%), SMN (91%) and SXT (9%). There was no resistance to CIP, TET, and ERY at the TCH. Eight (17%) isolates were from the TTH with the following resistance pattern: OX (13%), AMP (13%) and SMN (87%). There was no resistance to CIP, TET, SXT and ERY at the TTH. The details of the susceptibility testing results can be found as an Additional file 1.

### Multi-drug resistance pattern of the *S. aureus* isolates

From the 47 positive *S. aureus* isolates, nine (19%) were multi-drug resistant (resistant to three or more antibiotics), 11 (23%) were resistant to only two antibiotics, 23 (49%) were resistant to only one antibiotic and the remaining four (9%) showed no resistance to any of the antibiotics. Eight (89%) out of the nine multi- drug resistant *S. aureus* isolates were from TWH and one (11%) was from TCH. Nine (82%) of those resistant to only two antibiotics were from TWH while the remaining two (18%) were also from TTH. Ten (43%) of the isolates resistant to only one antibiotic were from TCH, eight (35%) were from TWH and five (22%) were from TTH. Out of the four that were not resistant to any antibiotic, three (75%) were from TWH and one (25%) was from TTH.

### Antibiotic resistance pattern of the MRSA isolates

**Fig. 2:**
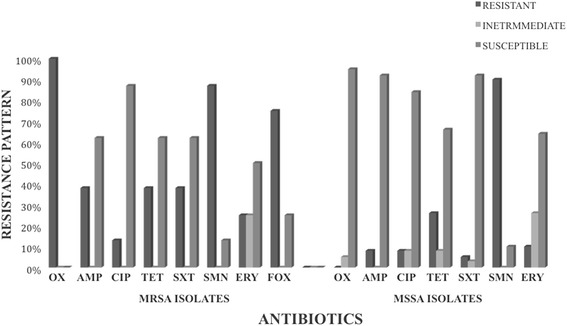
Resistance rates of Methicillin Susceptible *S. aureus* versus Methicillin Resistant *S. aureus.* TTH, Tamale Teaching Hospital, TCH, Tamale Central Hospital, TWH, Tamale West Hospital. Cefoxitin (FOX), Oxacillin (OX), Ciprofloxacin (CIP), Erythromycin (ERY), Tetracycline (TET), Ampicillin (AMP), Streptomycin (SMN) and Sulfamethoxazole-trimethoprim (SXT)

Of the total 47 (39%) positive *S. aureus* isolates, eight (17%) were Methicillin-resistant *S. aureus* (MRSA). The percentage resistance of the MRSA (eight isolates) to the antibiotics was OX (100%), AMP (38%), CIP (13%), TET (38%), SMN (87%), ERY, (25%), SXT (38%) and FOX (75%) (Fig. 2). Five (63%) of the MRSA isolates were multi-drug resistant and the other three (38%) were resistant to only two antibiotics. Seven of the MRSA isolates (87%) were from door handles and one (13%) was from a water tap in the TWH male ward. The MRSA resistant to all the antibiotics used was from a door handle from the TWH male’s ward urinal.

### Antibiotic resistance pattern of the MSSA isolates

Thirty-nine isolates (83%) of the total 47 positive *Staphylococcus* isolates were Methicillin-Susceptible *S. aureus* (MSSA). The rate of resistance of the 39 MSSA isolates to the antibiotics was: SMN (90%), TET (26%), ERY (10%), AMP (8%), CIP (8%) and SXT (5%) respectively (Fig. 2). Four (10%) of the MSSA were multi-drug resistant, nine (23%) were resistant to only two antibiotics, twenty-two (57%) were resistant to only one antibiotic and the remaining four (10%) were susceptible to all seven antibiotics. Of the four (10%) multi-drug resistant isolates, two (50%) were resistant to five antibiotics, one (25%) was resistant to four antibiotics and the remaining isolate (25%) was resistant to three antibiotics. Thirty-five of the MSSA isolates (90%) were resistant to Streptomycin (SMN). All the multi-drug resistant MSSA were from TWH. Three of the multi-drug resistant MSSA (75%) isolates were from door handles and the remaining isolate was from a theatre apron. Of the four multi-drug resistant MSSA isolates, the two that were resistant to five antibiotics were from the door handles of the theatre and the one resistant to four antibiotics was from the main entrance door handle of the male ward. The remaining isolate, resistant to three antibiotics, was from the door handle of the emergency ward at the OPD.

## Discussion

### MSSA and MRSA contamination of the hospital environment

This study was carried out to determine the prevalence and antibiotic resistance of *S. aureus* and MRSA isolates in the environments of three public hospitals in Ghana, and our results provide baseline information on *S. aureus* and MRSA prevalence in these hospital environments. Contamination of door handles with *S. aureus* occurred more at the administration blocks and OPD than the rest of the surfaces sampled; however, contamination recorded on the door handles and some of the surfaces in the operating theatres of these hospitals is clearly a concern. Although routine cleaning procedures are undertaken in these hospitals, they are not completely effective, and improved methods of disinfecting these hospital environments are recommended [17]. During the course of this study, it was observed that more attention was paid to cleaning floors rather than door handles or knobs, which may have allowed a build-up of *S. aureus* on these surfaces. Subsequently, the high contamination rate of door handles and surfaces by *S. aureus* in hospitals is likely to be a contributing factor for these bacteria being implicated in blood stream infections in Tamale [9] and other parts of Ghana [11, 13].

Preventive measures such as improved personal hygiene and the regular cleaning and disinfection of hospital door handles, stair rails and other points of contact are highly recommended, especially in the TWH, which recorded the highest *S. aureus* contamination rate among the three hospitals. The results of this study are similar to those of Newman [5] for a hospital in Accra where the *S. aureus* prevalence was 44% on contact surfaces at neonatal unit, and that of Hammuel et al. [6] for a hospital in Nigeria. Our study reports higher MRSA prevalence (87%) than a similar study in Brazil, which recorded 33.3% prevalence of *S. aureus* on hospital ward surfaces (bedside tables, bed rails and door handles) [18]. In a similar study by Oie et al. [19], 27% (versus 39% in our study) of the door handles in a University hospital in Japan were contaminated with *S. aureus* of which 20.9% (versus 83% in our study) were MSSA and 8.7% (17% in our study) were MRSA.

### Antibiotic resistance pattern

Antibiotic resistance has become a notorious health concern in the twenty-first Century. MRSA is an impediment to antimicrobial therapy, and the introduction of new classes of antimicrobial agents is usually followed by the emergence of resistant forms of this pathogen. The threat posed by MRSA was vividly demonstrated in this study, as one hospital isolate was resistant to all eight antibiotics used for the susceptibility test. Ghana is a place where poverty is prominent and people cannot afford to buy more expensive antibiotics, and the prevalence of MSRA on door handles, and particularly of theatre rooms, presents a significant threat to patients undergoing surgery.

Generally, the MSSA isolates in this study were more susceptible than the MRSA to antibiotics, but regular surveillance exercises should still be used to control the emergence of MRSA and other infectious diseases in hospitals. Resistance rates were fairly high for most of the antibiotics tested, although the pattern of resistance to Ciprofloxacin and Trimethoprim/sulfamethoxazole in MSRA isolates mean that these antibiotics can be recommended for the treatment of MRSA infections if antibiotics used in this study are the only available ones for treatment. These drugs also consistently demonstrate in vitro activity against *S. aureus* [4]. Hammuel et al. [6] also reported multi-drug resistance of *S. aureus* in the hospital environment in a similar study in Nigeria, but the multi-drug resistance of *S. aureus* in our study was lower (19% vs 31.30%).

## Conclusions

This is the first study conducted on *S. aureus* on hospital door handles, stair rails and other points of contact in Ghana. There were high levels of contamination of *S. aureus* and MRSA on door handles in some of the hospitals. Isolates of *S. aureus* and MRSA had high rates of resistance to the antibiotics used in this study including one isolate that was resistant to all antibiotics. There is a need for periodic surveillance and monitoring of *S. aureus* and MRSA in the hospital environment as well as regular and effective cleaning of hospital door rails and contact surfaces in Ghanaian hospitals. This study provides preliminary information for the control of infections in the three hospitals studied. Further studies are needed, not only in Ghana, but across Africa, to understand the extent to which *S. aureus* isolated from contact surfaces in the hospital is contributing to nosocomial diseases.

## Additional file

Additional file 1: Table S1. Summary of Antibiotic Susceptibility Results. (DOCX 33 kb)

## Abbreviations

AMP: Ampicillin
CIP: Ciprofloxacin
ERY: Erythromycin
FOX: Cefoxitin
MRSA: Methicillin resistant *Staphylococcus aureus*
MSSA: Methicillin susceptible *Staphylococcus aureus*
OPD: Out patients department
OX: Oxacillin
SMN: Streptomycin
SXT: Sulfamethoxazole-trimethoprim
TCH: Tamale Central Hospital
TET: Tetracycline
TTH: Tamale Teaching Hospital
TWH: Tamale West Hospital
