# Supplementary material for: Prevalence and pattern of antibiotic resistance of Staphylococcus aureus isolated from door handles and other points of contact in public hospitals in Ghana
Source: Antimicrob Resist Infect Control. 2017 May 10;6:44. doi: 10.1186/s13756-017-0203-2 (PMC5424397; doi:10.1186/s13756-017-0203-2)
Supplement: Additional file 1: Table S1. — Summary of Antibiotic Susceptibility Results. (DOCX 33 kb) [file 13756_2017_203_MOESM1_ESM.docx]

**Additional file 1: Table S2: Summary of Antibiotic Susceptibility Results**

| ***NO.*** | ***SAMPLE***  ***NO.*** | ***NAME OF SAMPLE***  ***(WHERE SAMPLE TAKEN)*** | ZONE DIAMETER BREAKPOINT | | ANTIBIOTICS, ZONE OF INHIBITION DIAMETER (D), RESISTANT(*®*), INTERMEDIATE (*ῗ* ) AND SUSCEPTIBILITY(*₰*) ON MUELLER-HINTON AGAR | | | | | | | | | | | | | | | |
| --- | --- | --- | --- | --- | --- | --- | --- | --- | --- | --- | --- | --- | --- | --- | --- | --- | --- | --- | --- | --- |
|  |  |  |  |  | ***OX, 1µg*** | | ***AMP, 25****µg* | | ***CIP, 5****µg* | | ***TET, 30****µg* | | ***SXT, 25****µg* | | ***SMN, 10****µg* | | ***ERY, 15****µg* | | ***FOX, 30µg*** | |
|  |  |  | ***® ≤*** | | **10** | | **14** | | **12** | | **14** | | **14** | | **23** | | **13** | | **22** | |
|  |  |  | ***≤ ῗ ≤*** | | **11-12** | | **-** | | **16-20** | | **15-18** | | **15-16** | | **-** | | **14-22** | | **-** | |
|  |  |  | **₰ ≥** | | **13** | | **15** | | **21** | | **19** | | **17** | | **24** | | **23** | | **23** | |
|  | 1 | OPD (CR 1 dh) |  |  | *25* | ₰ | *39* | ₰ | *29* | ₰ | *35* | ₰ | *35* | ₰ | *15* | *®* | *27* | ₰ |  |  |
|  | 2 | OPD (CR 2 dh) |  |  | *27* | ₰ | *31* | ₰ | *35* | ₰ | *32* | ₰ | *34* | ₰ | *25* | ₰ | *29* | ₰ |  |  |
|  | 3 | OPD (CR 5 dh) |  |  | *19* | ₰ | *33* | ₰ | *35* | ₰ | *19* | ₰ | *37* | ₰ | *21* | *®* | *32* | ₰ |  |  |
|  | 4 | OPD (CR 6 dh) |  |  | *19* | ₰ | *25* | ₰ | *34* | ₰ | *17* | *ῗ* | *31* | ₰ | *24* | ₰ | *39* | ₰ |  |  |
|  | 6 | OPD (Emergency ward bath dh) |  |  | *26* | ₰ | *27* | ₰ | *34* | ₰ | *9* | *®* | *27* | ₰ | *20* | *®* | *6* | *®* |  |  |
|  | 7 | OPD (Emergency ward toilet dh) |  |  | *19* | ₰ | *30* | ₰ | *30* | ₰ | *29* | ₰ | *31* | ₰ | *24* | ₰ | *28* | ₰ |  |  |
|  | 8 | OPD (Injection room dh) |  |  | *17* | ₰ | *30* | ₰ | *27* | ₰ | *29* | ₰ | *25* | ₰ | *18* | *®* | *23* | ₰ |  |  |
|  | 9 | OPD (Pharmacy dh) |  |  | *23* | ₰ | *24* | ₰ | *27* | ₰ | *29* | ₰ | *15* | *ῗ* | *18* | *®* | *27* | ₰ |  |  |
|  | 10 | OPD (Dressing room dh) |  |  | *17* | ₰ | *10* | *®* | *28* | ₰ | *26* | ₰ | *29* | ₰ | *21* | *®* | *24* | ₰ |  |  |
|  | 11 | OPD (Roller bed side handle) |  |  | *18* | ₰ | *28* | ₰ | *24* | ₰ | *8* | *®* | *25* | ₰ | *16* | *®* | *24* | ₰ |  |  |
|  | 12 | OPD (Roller bed mattress top) |  |  | *28* | ₰ | *27* | ₰ | *15* | *ῗ* | *9* | *®* | *25* | ₰ | *19* | *®* | *18* | *ῗ* |  |  |
|  | 13 | Administration (Registry 1^st^ dh) |  |  | *17* | ₰ | *29* | ₰ | *26* | ₰ | *23* | ₰ | *30* | ₰ | *22* | *®* | *27* | ₰ |  |  |
|  | 14 (mrsa) | Administration (Registry 2^nd^ dh) |  |  | *6* | *®* | *14* | *®* | *27* | ₰ | *21* | ₰ | *25* | ₰ | *19* | *®* | *24* | ₰ | 11 | *®* |
|  | 15 (mrsa) | Administration (Admin Office dh) |  |  | *6* | *®* | *18* | ₰ | *32* | ₰ | *8* | *®* | *28* | ₰ | *20* | *®* | *32* | ₰ | 21 | *®* |
|  | 16 | Administration (Stair rail) |  |  | *16* | ₰ | *22* | ₰ | *23* | ₰ | *28* | ₰ | *23* | ₰ | *14* | *®* | *19* | *ῗ* |  |  |
|  | 17 | X-RAY department inner dh |  |  | *16* | ₰ | *30* | ₰ | *30* | ₰ | *27* | ₰ | *30* | ₰ | *17* | *®* | *21* | *ῗ* |  |  |
|  | 18 | Theatre (Main entrance dh) |  |  | *17* | ₰ | *32* | ₰ | *22* | ₰ | *28* | ₰ | *26* | ₰ | *17* | *®* | *24* | ₰ |  |  |
|  | 19 | Theatre (Operation room dh) |  |  | *22* | ₰ | *35* | ₰ | *27* | ₰ | *8* | *®* | *29* | ₰ | *17* | *®* | *21* | *ῗ* |  |  |
|  | 20 | Theatre (Operation tap knob) |  |  | *15* | ₰ | *29* | ₰ | *25* | ₰ | *10* | *®* | *27* | ₰ | *17* | *®* | *22* | *ῗ* |  |  |
|  | 21 | Theatre (Bath dh) |  |  | *13* | ₰ | *25* | ₰ | *26* | ₰ | *12* | *®* | *30* | ₰ | *19* | *®* | *30* | ₰ |  |  |
|  | 24 | Theatre (Operation Apron 2) |  |  | *14* | ₰ | *23* | ₰ | *8* | *®* | *6* | *®* | *6* | *®* | *8* | *®* | *6* | *®* |  |  |
|  | 25 | Theatre (Dr. changing room 1dh near main entrance) |  |  | *14* | ₰ | *29* | ₰ | *6* | *®* | *6* | *®* | *6* | *®* | *6* | *®* | *6* | *®* |  |  |
|  | 27 | Theatre (Surgical ward dh) |  |  | *13* | ₰ | *14* | *®* | *29* | ₰ | *29* | ₰ | *18* | ₰ | *13* | *®* | *21* | *ῗ* |  |  |
|  | 28 | Male ward (Inner door phs) |  |  | *17* | ₰ | *16* | ₰ | *26* | ₰ | *9* | *®* | *21* | ₰ | *14* | *®* | *23* | ₰ |  |  |
|  | 29 | Male ward (Main entrance dh) |  |  | *17* | ₰ | *26* | ₰ | *6* | *®* | *6* | *®* | *23* | ₰ | *16* | *®* | *6* | *®* |  |  |
|  | 30 (mrsa) | Male Ward (Patients urinal dh) |  |  | *6* | *®* | *8* | *®* | *6* | *®* | *8* | *®* | *6* | *®* | *17* | *®* | *9* | *®* | 9 | *®* |
|  | 31 (mrsa) | Male Ward (Staff urinal dh) |  |  | *6* | *®* | *15* | ₰ | *30* | ₰ | *6* | *®* | *6* | *®* | *20* | *®* | *13* | *®* | 16 | *®* |
|  | 32 (mrsa) | Male Ward (Plastic hand washing tap knob) |  |  | *10* | *®* | *23* | ₰ | *22* | ₰ | *30* | ₰ | *23* | ₰ | *20* | *®* | *18* | *ῗ* | 32 | ₰ |
|  | 36 | Administration (Deputy Dir. of Nursing office dh) |  |  | *22* | *₰* | *32* | *₰* | *28* | *₰* | *22* | *₰* | *34* | *₰* | *17* | *®* | *28* | *₰* | TCH |  |
|  | 38 | Child Welfare Centre (Outer dh) |  |  | *21* | *₰* | *32* | *₰* | *26* | *₰* | *29* | *₰* | *38* | *₰* | *20* | *®* | *23* | *₰* |  |  |
|  | 41 | Lab (Microbiology unit dh) |  |  | *28* | *₰* | *38* | *₰* | *34* | *₰* | *34* | *₰* | *30* | *₰* | *17* | *®* | *32* | *₰* |  |  |
|  | 42 (mrsa) | Lab (Bleeding room dh) |  |  | *6* | *®* | *14* | *®* | *25* | *₰* | *22* | *₰* | *8* | *®* | *23* | *®* | *26* | *₰* | 20 | *®* |
|  | 44 | OPD (CR 2 dh) |  |  | *20* | *₰* | *34* | *₰* | *30* | *₰* | *30* | *₰* | *40* | *₰* | *20* | *®* | *30* | *₰* |  |  |
|  | 47 | OPD (CR 7 dh) |  |  | *16* | *₰* | *22* | *₰* | *40* | *₰* | *27* | *₰* | *36* | *₰* | *17* | *®* | *32* | *₰* |  |  |
|  | 52 | Fistula Theatre (changing room dh) |  |  | *14* | *₰* | *22* | *₰* | *25* | *₰* | *25* | *₰* | *26* | *₰* | *22* | *®* | *23* | *₰* |  |  |
|  | 55 | Fistula Department (Inner dh) |  |  | *14* | *₰* | *28* | *₰* | *23* | *₰* | *26* | *₰* | *24* | *₰* | *20* | *®* | *24* | *₰* |  |  |
|  | 57 (mrsa) | Fistula Department (Patients wash room dh) |  |  | *6* | *®* | *26* | *₰* | *28* | *₰* | *26* | *₰* | *26* | *₰* | *24* | *₰* | *20* | *ῗ* | 19 | *®* |
|  | 66 | Female ward (main entrance dh) |  |  | *16* | *₰* | *22* | *₰* | *23* | *₰* | *24* | *₰* | *24* | *₰* | *14* | *®* | *20* | *ῗ* |  |  |
|  | 67 | Female ward (Nurses rest room dh) |  |  | *13* | *₰* | *24* | *₰* | *27* | *₰* | *26* | *₰* | *26* | *₰* | *18* | *®* | *24* | *₰* |  |  |
|  | 73 | Administration (1^st^ floor North dh) |  |  | *15* | ₰ | *16* | ₰ | *19* | *ῗ* | *23* | ₰ | *25* | ₰ | *18* | *®* | *26* | ₰ | TH |  |
|  | 79 | Administration (2^nd^ floor stair rail) |  |  | *13* | ₰ | *13* | *®* | *24* | ₰ | *15* | *ῗ* | *24* | ₰ | *20* | *®* | *21* | *ῗ* |  |  |
|  | 81 | Administration (Down floor outer stair rail) |  |  | *15* | ₰ | *22* | ₰ | *24* | ₰ | *21* | ₰ | *25* | ₰ | *19* | *®* | *26* | ₰ |  |  |
|  | 97 | OPD (CR 3 dh, **A.0.17**) |  |  | *12* | *ῗ* | *21* | ₰ | *23* | ₰ | *21* | ₰ | *23* | ₰ | *21* | *®* | *18* | *ῗ* |  |  |
|  | 99 | OPD (CR 6 dh Medical specialist, **A.0.20**) |  |  | *20* | ₰ | *32* | ₰ | *29* | ₰ | *30* | ₰ | *40* | ₰ | *19* | *®* | *26* | ₰ |  |  |
|  | 109 | LAB (Biochemistry dh, **A.0.69)** |  |  | *26* | ₰ | *26* | ₰ | *32* | ₰ | *16* | *ῗ* | *30* | ₰ | *10* | *®* | *32* | ₰ |  |  |
|  | 111 | LAB (Dental dh, **A.0.24*)*** |  |  | *12* | *ῗ* | *17* | ₰ | *16* | *ῗ* | *21* | ₰ | *21* | ₰ | *27* | ₰ | *15* | *ῗ* |  |  |
|  | 117(mrsa) | Male Surgical Ward (main dh) |  |  | *7* | *®* | *23* | ₰ | *21* | ₰ | *20* | ₰ | *30* | ₰ | *19* | *®* | *25* | ₰ | 29 | ₰ |
